# Effects of Low-Temperature Stress during the Anther Differentiation Period on Winter Wheat Photosynthetic Performance and Spike-Setting Characteristics

**DOI:** 10.3390/plants11030389

**Published:** 2022-01-30

**Authors:** Yan Zhang, Lvzhou Liu, Xiang Chen, Jincai Li

**Affiliations:** 1 College of Agriculture, Anhui Agricultural University, Hefei 230036, China; zhangyan2022@stu.ahau.edu.cn (Y.Z.); liulvzhou@stu.ahau.edu.cn (L.L.); 2 Jiangsu Collaborative Innovation Centre for Modern Crop Production, Nanjing 210093, China

**Keywords:** *Triticum aestivum*, spring low-temperature stress, net photosynthetic rate, spikelets, grains

## Abstract

Climate change has caused frequent extreme low-temperature events to threaten global food security. Spring low-temperature stress is one of the major limiting factors for high and stable yields of wheat. We used two wheat varieties differing in spring cold-sensitivity (cold-tolerant variety Yannong 19 and cold-sensitive variety Xinmai 26) to examine the effects of low-temperature stress during the anther differentiation period on wheat photosynthetic performance and spike-setting characteristics. Low-temperature stress was simulated in a climate box at −2 °C, 0 °C or 2 °C (night) and 15 °C (day) for 24 h, 48 h or 72 h. With the extension of the treatment time and the decrease of temperature, the photosynthetic rate, stomatal conductance and transpiration rate of wheat leaves gradually decreased. All treatments except −2 °C for 72 h recovered slowly within 7–15 days after treatment. Low-temperature stress greatly reduced grains per spikelet, 1000-grain weight and yield per plant. By analyzing the spikelets in different stalk locations (upper, middle and lower), we found that the number of upper spikelets was significantly less than lower and middle spikelets after low-temperature stress. The sterile grain of upper spikelets (Xinmai 26, for example) can reach 100% at −2 °C for 48 h and 72 h, and the yield loss rate was 90.52% at 2 °C for 24 h, which was much higher than for the lower spikelets (60.73%) and middle spikelets (50.94%). Overall, these findings suggest that low-temperature stress during the anther differentiation period alters the photosynthetic activity involved in the accumulation of dry matter in wheat, which leads to delaying young spike growth, especially for upper spikelets, and ultimately in a decrease in yield.

## 1. Introduction

Wheat is one of the major food crops in the world—about 60 percent of the world’s population consumes wheat as a staple food [1]. The world population is expected to reach 9.73 billion in 2050, and crop production needs to more than double to meet demand, meaning wheat production needs to increase by at least 1/3 of the current growth rate [2,3]. Therefore, stable and high yields of wheat will be important challenges to address for ensuring global food security. Since the Industrial Revolution, global warming has been an indisputable fact. The United Nations Intergovernmental Panel on Climate Change (IPCC) Special Report on Climate Change and Land (SRCCL) pointed out that compared to 1850–1900, the global land average temperature from 2006 to 2015 has increased by about 1.53 °C, and it is expected to rise by more than 1.5 °C by 2100 [4]. Extreme weather caused by climate warming, such as low-temperature stress in spring, has posed a severe challenge to grain production [5]. Low-temperature stress has caused serious damage to wheat production in Australia [6,7,8,9], the United States [10], Europe [11] and China [12]. It is estimated that in Queensland and northern New South Wales, Australia, yield reductions due to spring low-temperature stress of winter cereals often causes 100% yield losses. Even under best management practices, low-temperature stress can reduce long-term average yields by 10% [7], and annual losses amounted to 100 million AUD [8]. From 1995 to 2010, 41 low-temperature frost events occurred in Kansas, USA, and the wheat yield reduction can reach 538 kg/hm^2^ in severe years [13]. In the past 40 years, nearly 20 large-scale low-temperature disasters [14,15] have occurred in major wheat producing areas in China. Low-temperature stress in spring was particularly prominent and more frequent in the Huang-huai wheat region, with events occurring in 2009, 2013, 2015, 2018 and 2020 that seriously affected the yield and quality of wheat [16].

Low-temperature stress in spring means that the temperature in the season of rebirth and growth (generally the end of February and the beginning of March in the northern hemisphere) rises faster, and in the late spring (generally the end of March to the beginning of April) the temperature is lower than normal. When the development of winter wheat enters the anther differentiation period, the young ears’ resistance to frost will drop sharply [17]. Once it encounters low-temperature stress, the differentiation of young ears will stop, resulting in fruitlessness or severe yield reduction, which can reduce production by 30–50% [12].

A large number of studies have shown that low-temperature stress at the booting stage can significantly reduce the photosynthetic rate of major functional leaves, such as the flag leaf and the second and third fully developed leaf [18,19], cause an accumulation of carbohydrates [20] and change endogenous hormone content and antioxidant enzyme activity [21,22]. However, few studies have documented the effects of low temperature stress on wheat during the anther differentiation period.

Photosynthesis is the source of plant material metabolism and energy transformation [23]. It is sensitive to abiotic stress, such as drought stress and high- and low-temperature stress [24]. Therefore, as an important indicator to judge the growth status and stress resistance of plants, photosynthetic characteristics are often used to reflect the degree to which the plant responds to stress [25]. Spring low-temperature stress adversely affects the growth and development of plants by affecting photosynthesis and then dry-matter accumulation and distribution; this leads to decreases in crop yield and quality [26].

In the present study, controlled phytotron experiments were conducted with different cold-sensitive winter wheat cultivars under different low-temperature levels and durations during the anther differentiation period. Our primary objectives were to (1) analyze the effects of low-temperature level and duration during the anther differentiation period on leaf photosynthetic properties, and (2) quantify the effects of low-temperature level and duration on spike-setting characteristics, especially wheat spikelets at different positions.

## 2. Materials and Methods

### 2.1. Experimental Design

Test materials: the cultivars Yannong 19 (cold-tolerant variety expressed by YN19) and Xinmai 26 (cold-sensitive variety expressed by XM26), as the main varieties cultivated in the Huang-huai wheat area (Table 1), were selected for experimentation on 22 October 2018 in the Agricultural Extraction Garden of Anhui Agricultural University (31°86′ N, 117°26′ E; 30 m altitude).

Test treatment: 0~20 cm tillage layer soil in the field (pH 6.5; organic matter 16.3 g·kg^−1^; available nitrogen 112.2 mg kg^−1^; available phosphorus 23.0 mg kg^−1^; available potassium 161.6 mg kg^−1^) was loaded into the test pots (30 cm diameter × 35 cm height; 3 drainage holes), which were then buried in the test field. Every pot was filled with 10 kg of soil; 11.52 g of compound fertilizer (N:P:K = 15:15:15) was applied to each pot before sowing, and 3 g of pure nitrogen was applied to each pot during the wheat jointing stage. 14 seeds were planted per pot, and 7 seedlings were reserved at the wheat trefoil stage.

From March to April 2019, after the regreening stage, 5 random spikes were collected every two days in order to observe the differentiation process under microscope (Olympus SZ2−ILST; Tokyo, Japan). When the young ears reached the anther differentiation period (50% of the wheat ears partly form a longitudinal split septum, dividing the anthers into four pollen bundles), all the pots except the control treatments (expressed by CK) were moved to the RXZ smart climate box (DGXM-1008; Ningbo Jiangnan Instrument Manufacturing Factory, Ningbo, China; 1300 mm length × 630 mm width × 1305 mm height) with humidity 70% and light 17,000 LX during the day and 0 LX at night for treatment (Table 2).

### 2.2. Plant Measurement and Methods

Determination of photosynthetic parameters: the second fully developed leaf from the growing point was selected, and the photosynthetic indexes were measured by using a LI-6400 (LI-COR, Lincoln, NE, USA) portable photosynthesis system; relative chlorophyll content (SPAD) was measured with a SPAD-502 (Konica Minolta, Tokyo, Japan).

Photosynthetic indexes measured were: net photosynthetic rate (Pn); transpiration rate (Tr); stomatal conductance (Gs); and intercellular CO_2_ concentration (Ci).

The wheat was harvested at maturity, and the yield factor was calculated by recording the number of spikes per plant, number of grains per spikelet, 1000-grain weigh and yield per plant.

### 2.3. Plant Sampling

After low-temperature treatment, wheat leaves were selected to measure four photosynthetic indexes. Each treatment was repeated 3 times. At maturity, 3 plants were randomly selected from each treatment in order to assess the number of spikes per plant, grains per spike, grain yield per plant, and yield for the upper, middle and lower regions (6–7 spikelets per region). All pots were used for the 1000-grain weight.

### 2.4. Data Processing

Data were subjected to analysis of variance (univariate, two-way ANOVA and three-way ANOVA), and statistical divergence among treatments was determined using Duncan’s new multiple range test. Correlation tests were calculated using KMO (Kaiser–Meyer–Olkin). Statistical analyses were conducted using SPSS statistical software (version 20; SPSS, Inc., Chicago, IL, USA).

## 3. Results

### 3.1. Photosynthetic Parameters of Wheat

#### 3.1.1. Photosynthetic Parameters Relative to Variety, Treatment Temperature and Time

The three-factor variance analysis of varieties, treatment temperature and time of various photosynthetic parameters showed that the photosynthetic parameters and the three-factor interaction were extremely significant 3 days after treatment, and then the correlation gradually weakens. The correlation was significant again at 15 days after treatment. (Table 3).

#### 3.1.2. Changes to Photosynthetic Parameters of Wheat after Treatment

##### Net Photosynthetic Rate

**Figure 1:**
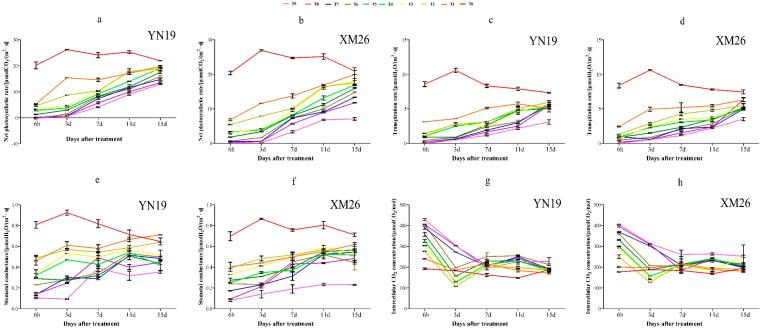
Photosynthetic indices under different low−temperature treatments during the anther differentiation period. (**a**) Net photosynthetic rate changes of cold−tolerant variety. (**b**) Net photosynthetic rate changes of cold−sensitive variety. (**c**) Transpiration rate changes of cold−tolerant variety. (**d**) Transpiration rate changes of cold−sensitive variety. (**e**) Stomatal conductance changes of cold−tolerant variety. (**f**) Stomatal conductance changes of cold−sensitive variety. (**g**) Intercellular CO_2_ concentration of cold−tolerant variety. (**h**) Intercellular CO_2_ concentration of cold−sensitive variety. Data represent means ± SE (*n* = 3).

Figure 1a,b indicates that under the same low-temperature treatment level, the Pn of the two wheat varieties showed a downward trend with the extension of the low temperature, and the leaf Pn of the two wheat cultivars decreased to the lowest 6 h–3 days after treatment. Compared with CK, the value for YN19 and XM26 decreased by 69.3% and 75.3%, respectively. Three days after low-temperature treatment, the leaf Pn of the two wheat cultivars began to rise, and the 24 h treatments were able to return to the control level within 15 days. However, the treatments of 48 h and 72 h were still lower than CK by varying degrees after 15 days. The Pn of XM26 leaves treated at −2 °C for 72 h no longer increased after a 3–11-day recovery period—they maintained a low level. In addition, the speed of recovery of YN19 leaf Pn was better than that of XM26 after low-temperature treatment.

##### Transpiration Rate

Under the same low-temperature level, but with the extension of the low-temperature duration, the leaf Tr of the two wheat varieties showed a decreasing trend (Figure 1c,d), while under the 72 h treatment, the leaf Tr of the two wheat varieties decreased first and then increased. Fifteen days after treatment, the Tr of wheat leaves treated at 24 h and 48 h could basically recover to the control level. However, the two varieties treated at −2 °C for 72 h failed to recover to the control level within 15 days. In addition, the low-temperature treatment level had a greater impact on YN19 blades than XM26.

##### Stomatal Conductance

The Gs of the two wheat cultivars under low-temperature treatment was basically consistent with the Tr (Figure 1e,f). With the extension of the low-temperature duration, Gs first decreased and then increased. For 72 h at −2 °C, the Gs of YN19 and XM26 leaves were only 12.4% and 15.7% that of CK, respectively. For the 24 h low-temperature treatment, the Gs of wheat leaves basically recovered to the control level. Both YN19 and XM26 Gs decreased with the decrease of temperature, with the influence of low-temperature treatment on XM26 greater than that of YN19.

##### Intercellular CO_2_ Concentration

In the control treatment, the Ci of the two varieties was not much different—both were about 200 μmolCO_2_·mol^−1^. With the decrease of temperature and the extension of treatment time, Ci had an increasing trend (Figure 1g,h).

### 3.2. Relative Chlorophyll Content (SPAD) of Wheat

**Figure 2:**
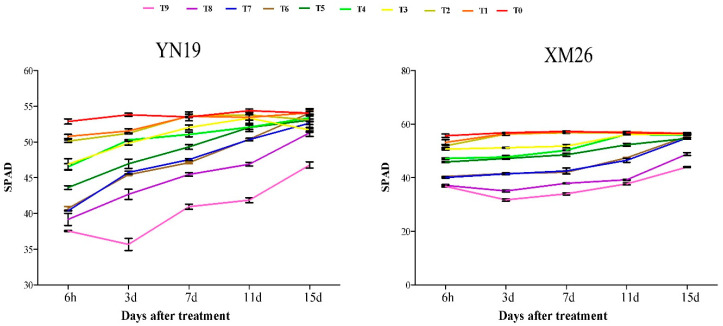
SPAD under different recovery times after low−temperature treatment during the anther differentiation stage. Data represent means ± SE (*n* = 3).

SPAD of 72 h treatment leaves was significantly lower than others up to 15 days after treatment (Figure 2). Except for the −2 °C 72 h treatment group, SPAD values gradually increased after 3 days, and the rate of recovery after 11 days was faster; the 24 h and 48 h treatments gradually returned to normal starting 1 days after treatment. In addition, the SPAD recovery ability of YN19 is significantly stronger than XM26.

### 3.3. Wheat Spike-Setting Characteristics

#### 3.3.1. Spike-Setting Characteristics and Yield Relative to Variety, Temperature Treatment, Treatment Time

The response to the temperature treatments varied depending on the position of the wheat spikes. Variety, treatment temperature and treatment time all had a significant effect on the upper-spikelet grain number.

The three factors had extremely significant effects on the 1000-grain weight of the wheat. The yield of the upper spikelets was greatly reduced in both varieties and at each treatment time. (Table 4).

#### 3.3.2. Changes of Spike-Setting Characteristics

Low-temperature stress during the anther differentiation period affected the number of grains per spike. Some spikelets and florets were damaged, resulting in a delay or even cessation of normal differentiation and development. Ultimately, yield was reduced. With the decrease of the treatment temperature and the extension of the stress time, the number of grains per spike decreased, and grain-setting loss rate increased—the difference between the treatments was significant.

With the decrease of the treatment temperature and the increase of the treatment time, the number of grains showed a decreasing trend from the middle spikelets to the upper and the lower spikelets. The upper spikelet grain-setting loss rate was the largest. (Table 5) Cultivars and treatments had significant effects on the number of grains per spikelet for the upper spikelets (Table 4).

Low-temperature stress during the anther differentiation period has a great influence on wheat yield per plant, which is positively correlated with the intensity of treatment. The rate of spikelet setting and 1000-grain weight were decreased.

Under low-temperature stress, the yield-reduction range at different positions of the wheat spike varied. The yield decrease of upper spikelets was much higher than lower and middle spikelets. The degradation of upper spikelets was the main cause of yield reduction (Table 6).

### 3.4. The Relationship between Photosynthetic Parameters and Wheat Spike-Setting Characteristics

The Pn of leaves after low-temperature treatment has a significant positive correlation with the grains per spike and the yield. The correlation weakens with the passage of time after treatment.

The correlation between Pn and 1000-grain weight of XM26 has an extremely significant correlation after treatment, which is different from YN19 (Table 7).

## 4. Discussion

Photosynthesis is one of the physiological processes most sensitive to low temperature. A number of studies have implicated the effects of low temperature stress on photosynthesis of wheat leaves [29,30]. However, previous studies mostly focused on the effects of low-temperature levels on wheat leaf photosynthesis, or on the effects of low-temperature duration on leaf photosynthesis, and less consideration of the comprehensive regulation effects of low-temperature levels and duration. This study showed that low temperature significantly reduces the Pn of wheat leaves, which is consistent with previous studies. Under the same low-temperature duration, the Pn of wheat leaves decreased with the decrease of temperature. At the same low-temperature level, Pn of YN19 and XM26 dropped to the lowest value 6 h–3 days after treatment, and then began to rise. The reason may be that the short-term low-temperature adaptation increases the main enzymes content in the leaf photosynthesis process, promoting the photosynthetic index and enhancing the stability of the cell membrane, thereby alleviating the effect of low temperature stress on leaf photosynthesis to a certain extent. However, with the low-temperature treatment at −2 °C for 72 h, the Pn of wheat leaves showed a downward trend with the decrease of temperature and the extension of the low-temperature duration, and it could not return to the normal value. This phenomenon is more obvious in the XM26 variety. Guan et al. [31] studied the effects of different genotypes of wheat variety on photosynthetic performance under low-temperature stress and found that, after low-temperature stress at the jointing stage, the photosynthetic performance of semi-winter variety YN19 was higher than that of the feeble-spring variety Zhengmai9023 and the spring variety Yangmai18, indicating that YN19 has a higher photosynthetic activity and a stronger self-protection mechanism. Marcellos [32] revealed that the Pn of leaves under mild low-temperature stress can be restored to normal levels 3 days after treatment. However, the Pn of leaves under severe low-temperature stress cannot be restored. Fuller et al. [33] reported that, after the booting stage, the wheat flag leaves were damaged at −5 °C. When the temperature was lower than −5 °C, the wheat flag leaves were seriously damaged and the Pn of leaves was hard to recover to a normal level. These views are consistent with the results of our study.

The decrease of Pn caused by low temperature is accompanied by the decrease of Gs [34]. Similar results were observed in this experiment. During low-temperature treatment, the Pn of YN19 and XM26 leaves showed a downward trend with the extension of low temperature. Under the same low-temperature duration, wheat leaf Pn, Gs and Tr all showed a decreasing trend with the decrease of temperature, while Ci increased significantly with the decrease of temperature. These results indicate that low temperature causes a decrease in the Pn of wheat leaves, reduces wheat dry-matter accumulation and ultimately leads to a decline in yield. After the low-temperature treatment, except for the −2 °C 72 h treatment, the Pn, Gs, Tr and SPAD of the wheat leaves basically returned to the control level. It can be preliminarily determined that −2 °C 72 h is the threshold for wheat death as a result of low-temperature stress during the anther differentiation period. For below-zero low-temperature stress, the chlorophyll content and photosynthetic rate of wheat leaves decreases significantly. The cold-tolerant wheat has a strong self-protection mechanism: the photosynthetic organs are less damaged and they have high photosynthetic activity.

This study showed that low-temperature stress during the anther differentiation period can reduce the number of spikes per plant and the number of grains per spike. The decrease in grain number per spike is due to the changes in photosynthesis, which affects dry-matter accumulation [35]. Low-temperature stress during the anther differentiation period affects the normal floret development by restricting photosynthate distribution in the spikes. Ke et al. [36] found that low-temperature stress in wheat during the anther connective stage causes significant reduction in dry-matter accumulation. Prior to anthesis, it severely disrupts the transport efficiency, which results in uneven distribution of grain dry-matter content. Grain dry-matter content’s uneven distribution caused shorter, pinched and reduced number of grains per spike, which is the main cause of wheat yield. We found that the low-temperature stress during the anther differentiation period mainly caused the young wheat spikes to freeze, causing some stems and tillers to freeze to death, and the number of spikes was significantly reduced. Some spikelets were frozen, and the differentiation process of new-born tillers was shortened, and the number of grains in the final spike was significantly reduced. Yun et al. [37] also reported similarly that low-temperature stress in spring can lead to a decrease in the number of effective spikes and grains per spike, resulting in reduced yields.

Low-temperature stress during the anther differentiation period not only reduced the number of grains per spike but also 1000-grain weight. Xiao et al. [38] analyzed that the reason for the reduction of wheat yield caused by low temperature in spring was treatment at elongation and booting stages, which damaged the functional leaves, affected the synthesis and supply of organic matter, and caused a significant decrease in 1000-grain weight. Low-temperature stress during the anther differentiation period weakened photosynthesis of the main functional leaves of the wheat, resulting in changes of dry-matter accumulation, translocation and distribution. This change resulted in the decrease of grain number per spike and the 1000-grain weight, which ultimately had a great impact on the decrease of yield [39]. Additionally, low-temperature stress during the anther differentiation period had different effects based on spikelet position. Damage was much more serious to the upper spikelets than to the lower spikelets, while the damage to the lower spikelets was slightly higher than to the middle spikelets. The yield reduction of upper spikelets, which was caused by their reduction of grain number, was the main reason for the yield reduction of whole spike.

In conclusion, low-temperature stress affects the relationship between photosynthetic performance and spike-setting characteristics which means making the source–sink relationship of wheat unbalanced [40], finally resulting in a substantial decrease in yield. Sink and source interact and promote, but also oppose each other. Regarding the growth process of wheat, the differentiation process of young spikes at the jointing stage is accelerated [41], so that the sink develops rapidly, the leaf area per plant increases, the leaf area index increases and the source–sink balance relationship is established [42]. After the booting, in order to meet the continuous expansion of sink capacity, the leaf area per plant and the leaf area index are further improved, the total storage capacity is determined after heading, the source leaves give full play to photosynthetic performance, and the transportation and distribution of photosynthetic compounds from the source to the storage are coordinated [43]. This coordinated relationship between source and sink flows needs to maintain balance for a long time. Large source and strong sink are one of the conditions necessary for achieving high yield and quality of wheat, but the regulation of source and sink on wheat yield and quality is often restricted. Only based on a certain leaf and grain quality balance can a high-quality source-sink balance relationship be established, which helps to achieve the goal of high yield and quality of wheat.

## 5. Conclusions

Under the conditions of climate warming, cultivating and selecting varieties resistant to low-temperature stress in spring is an important way for wheat to resist spring low temperature and to provide a stable yield. The results of our experiment showed that low-temperature stress during anther differentiation has serious effects on photosynthesis, spike-setting characteristics and yield of wheat. In the breeding and identification of varieties, photosynthetic indicators, number of grains per spike, etc., can be used to evaluate the frost tolerance of wheat reference indicators in order to focus on breeding low temperature stress resistant variety. The results also preliminarily showed that the low-temperature stress during anther differentiation especially effects the upper spikelets of wheat—the difference in yield among varieties was mainly due to the difference in seed setting of upper spikelets. Low-temperature stress during anther differentiation affects the grain-setting characteristics of wheat mainly through affecting the photosynthetic characteristics of leaves.

In future work, the physiological and molecular mechanisms affecting spike development and grain establishment in wheat will be explored, thus providing support for disaster prevention and mitigation of wheat production.

## Figures and Tables

**Table 1:** Information about the two cultivars.

Cultivar	Breeding Unit	Certification Unit	Information
Yannong 19	Yantai Academy of Agricultural Sciences, Shandong Province, China	Shandong Provincial Crop Variety Certification Committee, China	Medium-gluten winter wheat variety. The main wheat variety planted in Anhui Province [27].
Xinmai 26	Xinxiang Academy of Agricultural Sciences, Henan Province, China	China National Crop Variety Examination and Approving Committee	Super stringy semi-winter wheat variety. Suitable for planting in the south of Huang-huai winter wheat area, China. Attention should be paid the pre-vention of spring low-temperature stress after sowing [28].

**Table 2:** Test treatment.

Treatment	Day/Night Temperature (°C)	Day/Night Time (h)
T0(CK)	15/15	
T1	15/2	12/12 (1 day)
T2	15/2	12/12 (2 days)
T3	15/2	12/12 (3 days)
T4	15/0	12/12 (1 day)
T5	15/0	12/12 (2 days)
T6	15/0	12/12 (3 days)
T7	15/−2	12/12 (1 day)
T8	15/−2	12/12 (2 days)
T9	15/−2	12/12 (3 days)

The average daily temperature during the treatment was 15 °C.

**Table 3:** ANOVA of photosynthetic parameters relative to variety, treatment temperature and duration.

	**Pn**						**Tr**				
**Source**	**df**	**6 h**	**3 days**	**7 days**	**11 days**	**15 days**	**6 h**	**3 days**	**7 days**	**11 days**	**15 days**
Variety (V)	1	5.061 *	24.679 **	1.742	23.700 **	0.702	1.754	5.150 *	4.459 *	26.250 **	212.654 **
Time (T)	2	107.372 **	4817.742 **	180.756 **	272.750 **	71.056 **	72.772 **	400.596 **	144.267 **	148.309 **	64.425 **
TEMP	3	2094.333 **	46,721.242 **	1876.492 **	919.008 **	143.510 **	2689.090 **	5683.113 **	1180.115 **	593.546 **	396.052 **
V × T	2	0.294	67.738 **	0.211	1.319	14.917 **	3.563 *	1.841	7.468 **	3.597 *	17.443 **
V × TEMP	3	0.385	58.008 **	2.474	1.95	2.034	0.455	3.651 *	0.238	2.51	45.499 **
T × TEMP	6	14.837 **	970.723 **	27.565 **	34.058 **	8.570 **	16.031 **	58.279 **	20.988 **	19.242 **	10.964 **
V × T × TEMP	6	0.491	30.416 **	0.155	0.738	2.770 *	1.919	6.823 **	2.494 *	2.128	15.581 **
	**Gs**						**Ci**				
**Source**	**df**	**6 h**	**3 days**	**7 days**	**11 days**	**15 days**	**6 h**	**3 days**	**7 days**	**11 days**	**15 days**
Varieties (V)	1	23.092 **	256.971 **	6.766 *	0.79	0.564	217.686 **	1974.806 **	0.562	4.692 *	6.934 *
Time (T)	2	134.894 **	200.395 **	36.961 **	39.917 **	20.439 **	1590.326 **	48,057.871 **	0.989	114.223 **	1.196
TEMP	3	553.162 **	719.595 **	205.825 **	105.302 **	42.544 **	2772.767 **	1652.910 **	19.683 **	195.804 **	8.332 **
V × T	2	0.834	7.642 **	4.161 *	0.521	2.979	4.764 *	47.854 **	2.829	0.75	0.164
V × TEMP	3	4.410 **	12.159 **	0.782	5.186 **	2.123	3.665 *	166.138 **	1.728	4.434 **	0.576
T × TEMP	6	15.926 **	23.716 **	9.296 **	10.376 **	5.653 **	188.097 **	7786.114 **	1.13	13.244 **	0.669
V × T × TEMP	6	1.065	4.407 **	3.918 **	1.288	2.851 *	5.384 **	50.951 **	3.708 **	6.360 **	0.643

Note: * indicate significance at the level of 0.05, ** indicates significance at the level of 0.01.

**Table 4:** ANOVA of spike-setting characteristics and yield relative to variety, treatment temperature and treatment time.

Source	df	Grains per Spike	Grains per Upper Spikelet	Grains per Middle Spikelet	Grains per Lower Spikelet	Yield per Plant (g)	1000−Grain Weight	Upper Spikelet Yield (g)	Middle Spikelet Yield (g)	Lower Spikelet Yield (g)
Variety (V)	1	5.646 *	5.608 *	481.909 **	3.354	51.246 **	1645.898 **	6.806 *	0.515	2.472
Time (T)	2	187.029 **	136.636 **	63.950 **	7.805 **	56.403 **	2729.459 **	60.831 **	153.725 **	26.966 **
TEMP	3	1834.842 **	1203.659 **	610.441 **	339.778 **	2275.089 **	1100.179 **	261.209 **	537.149 **	1889.350 **
V × T	2	6.557 **	10.399 **	10.297 **	0.094	7.921 **	1117.622 **	4.474 *	6.626 **	1.131
V × TEMP	3	2.804 *	2.880 *	1.177	1.925	12.260 **	90.969 **	3.254 *	6.026 **	8.190 **
T × TEMP	6	24.301 **	16.326 **	7.532 **	4.590 **	7.963 **	346.694 **	8.733 **	18.726 **	5.529 **
V × T × TEMP	6	1.533	1.319	1.652	0.491	1.264	154.425 **	1.038	1.287	0.262

Note: * indicate significance at the level of 0.05; ** indicates significance at the level of 0.01.

**Table 5:** Spike-setting characteristics among different temperature treatments (compared with the CK of the same variety) after harvest.

Variety	Treatment	Grains per Spike	Grain−Setting Loss Rate	Grains per Upper Spikelet	Upper Spikelet Grain−Setting Loss Rate	Grains per Middle Spikelet	Middle Spikelet Grain−Setting Loss Rate	Grains per Lower Spikelet	Lower Spikelet Grain−Setting Loss Rate
YN19	72 h −2 °C	3.40 ± 1.00 e	52.02%	0.00 ± 0.00 g	100.00%	1.40 ± 1.14 g	92.55%	2.00 ± 2.35 bcd	80.00%
	72 h 0 °C	4.00 ± 0.00 e	51.26%	0.00 ± 0.00 g	100.00%	4.00 ± 0.71 f	78.72%	0.00 ± 0.00 f	100.00%
	72 h 2 °C	4.20 ± 0.58 e	51.01%	0.00 ± 0.00 g	100.00%	4.20 ± 1.63 f	77.66%	0.00 ± 0.00 f	100.00%
	48 h −2 °C	9.60 ± 0.58 d	44.19%	0.00 ± 0.00 g	100.00%	7.80 ± 2.95 cde	58.51%	0.60 ± 0.55 def	94.00%
	48 h 0 °C	9.20 ± 0.00 d	44.70%	1.20 ± 0.84 fg	91.30%	7.40 ± 0.55 de	60.64%	1.80 ± 1.64 bcdef	82.00%
	48 h 2 °C	13.80 ± 0.58 c	38.89%	1.40 ± 0.89 fg	89.86%	10.00 ± 1.22 c	46.81%	2.40 ± 2.51 bc	76.00%
	24 h −2 °C	14.80 ± 1.00 c	37.63%	4.60 ± 1.52 cd	66.67%	9.00 ± 0.71 cde	52.13%	1.20 ± 1.1 bcdef	88.00%
	24 h 0 °C	20.20 ± 1.00 b	30.81%	5.60 ± 1.52 bc	59.42%	12.00 ± 1.41 b	36.17%	2.60 ± 1.52 b	74.00%
	24 h 2 °C	21.40 ± 0.58 b	29.29%	6.60 ± 1.34 b	52.17%	12.40 ± 0.89 b	34.04%	2.40 ± 0.89 bc	76.00%
	ck	44.60 ± 0.58 a		13.80 ± 0.84 a		18.80 ± 1.30 a		10.00 ± 1.87 a	
XM26	72 h −2 °C	3.00 ± 0.00 f	53.79%	0.00 ± 0.00 g	100.00%	3.00 ± 0.71 fg	84.38%	0.00 ± 0.00 f	100.00%
	72 h 0 °C	4.00 ± 1.00 f	52.53%	0.00 ± 0.00 g	100.00%	4.00 ± 1.00 f	79.17%	0.00 ± 0.00 f	100.00%
	72 h 2 °C	4.00 ± 1.00 f	52.53%	0.00 ± 0.00 g	100.00%	4.00 ± 1.00 f	79.17%	0.00 ± 0.00 f	100.00%
	48 h −2 °C	8.00 ± 1.00 e	47.47%	0.60 ± 1.34 fg	95.77%	7.20 ± 3.70 e	62.50%	0.20 ± 0.45 ef	97.96%
	48 h 0 °C	9.80 ± 0.58 de	45.20%	1.20 ± 0.84 fg	91.55%	7.80 ± 0.83 cde	59.38%	0.80 ± 0.84 cdef	91.84%
	48 h 2 °C	14.00 ± 0.58 bc	39.90%	1.20 ± 0.84 fg	91.55%	10.00 ± 1.58 c	47.92%	2.80 ± 0.84 b	71.43%
	24 h −2 °C	12.40 ± 1.00 cd	41.92%	2.80 ± 1.1 e	80.28%	9.00 ± 1.41 cde	53.13%	0.60 ± 0.89 def	93.88%
	24 h 0 °C	15.80 ± 0.58 b	37.63%	3.60 ± 0.89 de	74.65%	9.60 ± 1.34 cd	50.00%	1.40 ± 0.55 bcdef	85.71%
	24 h 2 °C	15.00 ± 0.58 bc	38.64%	3.80 ± 1.3 de	73.24%	9.80 ± 2.28 c	48.96%	2.60 ± 1.14 b	73.47%
	ck	45.60 ± 0.58 a		14.20 ± 1.3 a		19.20 ± 1.10 a		9.80 ± 1.79 a	

Data represent mean ± SE (*n* = 3). Different letters following the data within each column mean significant difference at *p* ≤ 0.05.

**Table 6:** Yield among different temperature treatments (compared with CK) of the same variety after harvest.

Variety	Treatment	1000−Grain Weight (g)	Yield per Plant (g)	Yield per Plant Yield Reduction Rate (%)	Upper Spikelets Yield (g)	Middle Spikelets Yield (g)	Lower Spikelets Yield (g)	Upper Spikelets Yield Reduction Rate (%)	Middle Spikelets Yield Reduction Rate (%)	Lower Spikelets Yield Reduction Rate (%)
YN19	72 h −2 °C	35.16 ± 0.30 h	0.05 ± 0.08 h	99.53	0.00 ± 0.00 f	0.05 ± 0.04 i	0.07 ± 0.08 fg	100.00	94.10	84.00
	72 h 0 °C	35.97 ± 0.64 g	0.07 ± 0.13 h	99.44	0.00 ± 0.00 f	0.14 ± 0.02 h	0.00 ± 0.00 g	100.00	82.77	100.00
	72 h 2 °C	36.01 ± 0.00 g	0.20 ± 0.09 h	98.22	0.00 ± 0.00 f	0.15 ± 0.05 h	0.00 ± 0.00 g	100.00	81.88	100.00
	48 h −2 °C	39.60 ± 0.15 f	0.45 ± 0.12 gh	92.54	0.00 ± 0.00 f	0.31 ± 0.1 g	0.05 ± 0.04 fg	100.00	63.00	88.14
	48 h 0 °C	43.42 ± 0.15 e	0.41 ± 0.03 gh	90.59	0.03 ± 0.02 def	0.32 ± 0.02 fg	0.07 ± 0.07 fg	95.70	61.51	83.78
	48 h 2 °C	45.00 ± 0.01 c	1.08 ± 0.22 def	80.50	0.06 ± 0.04 d	0.45 ± 0.05 d	0.11 ± 0.11 ef	89.61	46.09	75.42
	24 h −2 °C	45.52 ± 0.30 b	1.15 ± 0.50 def	84.13	0.06 ± 0.04 de	0.41 ± 0.03 def	0.21 ± 0.07 cd	90.99	50.93	52.34
	24 h 0 °C	46.99 ± 0.01 a	1.43 ± 0.39 cd	66.47	0.12 ± 0.06 c	0.56 ± 0.06 c	0.26 ± 0.07 bc	79.85	32.45	40.11
	24 h 2 °C	47.05 ± 0.06 a	1.79 ± 0.10 c	60.48	0.11 ± 0.04 c	0.58 ± 0.04 c	0.31 ± 0.06 b	81.38	30.12	29.33
	ck	43.94 ± 0.09 d	6.79 ± 0.33 a		0.61 ± 0.03 b	0.83 ± 0.05 b	0.44 ± 0.08 a			
XM26	72 h −2 °C	41.41 ± 0.58 h	0.05 ± 0.09 h	99.59	0.00 ± 0.00 f	0.12 ± 0.03 hi	0.00 ± 0.00 g	100.00	86.40	100.00
	72 h 0 °C	43.00 ± 0.00 f	0.05 ± 0.09 h	99.43	0.00 ± 0.00 f	0.17 ± 0.04 h	0.00 ± 0.00 g	100.00	81.17	100.00
	72 h 2 °C	42.00 ± 0.06 g	0.17 ± 0.147 h	98.89	0.00 ± 0.00 f	0.17 ± 0.04 h	0.00 ± 0.00 g	100.00	81.61	100.00
	48 h −2 °C	42.70 ± 0.46 f	0.34 ± 0.32 gh	97.75	0.01 ± 0.02 ef	0.31 ± 0.14 g	0.03 ± 0.06 g	98.74	66.35	94.51
	48 h 0 °C	43.42 ± 0.15 e	0.70 ± 0.39 fg	92.99	0.04 ± 0.03 def	0.34 ± 0.32 efg	0.05 ± 0.04 fg	94.86	62.92	88.82
	48 h 2 °C	44.63 ± 0.12 c	0.96 ± 0.56 def	87.66	0.05 ± 0.03 def	0.45 ± 0.06 d	0.13 ± 0.04 ef	92.07	51.14	73.20
	24 h −2 °C	43.63 ± 0.00 e	0.39 ± 0.35 gh	92.88	0.03 ± 0.03 def	0.39 ± 0.05 defg	0.12 ± 0.05 ef	96.13	57.01	73.80
	24 h 0 °C	44.22 ± 0.00 d	0.76 ± 0.07 efg	88.50	0.12 ± 0.05 c	0.42 ± 0.05 de	0.16 ± 0.04 de	82.98	53.53	65.86
	24 h 2 °C	45.73 ± 0.01 b	1.23 ± 0.23 de	79.67	0.06 ± 0.02 d	0.45 ± 0.09 d	0.17 ± 0.06 de	90.52	50.94	62.73
	ck	47.58 ± 0.11 a	5.75 ± 0.07 b		0.68 ± 0.06 a	0.91 ± 0.05 a	0.45 ± 0.09 a			

Note: Data represent means ± SE (*n* = 3). Data represent mean ± SE (*n* = 3). Different letters following the data within each column mean significant difference at *p* ≤ 0.05.

**Table 7:** The correlation coefficients between Pn and wheat spike-setting characteristics.

			Pn (6 h)	Pn (3 d)	Pn (7 d)	Pn (11 d)	Pn (15 d)
R	Grains per spike	YN19	0.971 **	0.969 **	0.9773 **	0.9606 **	0.8835 **
		XM26	0.9875 **	0.9744 **	0.9667 **	0.9355 **	0.7255 *
	1000−grain weight	YN19	0.8858 **	0.9288 **	0.5585	0.695 *	0.8825 **
		XM26	0.9044 **	0.9203 **	0.9218 **	0.9359 **	0.8662 **
	Yield	YN19	0.9965 **	0.9453 **	0.9656 **	0.907 **	0.7703 **
		XM26	0.9784 **	0.9625 **	0.9523 **	0.8547 **	0.6112

Note: Critical value of correlation coefficient * *p* ≤ 0.05; ** *p* ≤ 0.01.

## Data Availability

Data is contained within the article.
